# Case Report: Cervical cancer masquerading as ovarian tumor: diagnostic challenges in a case with severe pyometra-pyocolpos complex

**DOI:** 10.3389/fonc.2025.1647366

**Published:** 2025-08-27

**Authors:** Chaoping Xie, Hongcen Liu, Daxia Huang, Ganmei Zhao, Min Qiu, Yong Liu, Dongwei Mao

**Affiliations:** ^1^ Department of Gynecology, Shenzhen Hospital of Guangzhou University of Chinese Medicine, Shenzhen, Guangdong, China; ^2^ Department of Radiology, Shenzhen Hospital of Guangzhou University of Chinese Medicine, Shenzhen, Guangdong, China; ^3^ Department of Gynecology, Guang’anmen Hospital China Academy of Chinese Medical Sciences, Beijing, China

**Keywords:** cervical carcinoma, pyometra, vaginal adhesions, case report, postmenopausal women, squamous cell carcinoma, misdiagnosis

## Abstract

**Introduction and importance:**

Early-stage cervical cancer, which is often asymptomatic, presents considerable diagnostic difficulties when accompanied by vaginal adhesions that conceal malignant lesions. In this report, we describe an exceptionally rare case of cervical cancer complicated by extensive pyometra and pyocolpos, highlighting key diagnostic challenges and evidence-based treatment approaches.

**Case presentation:**

A 61-year-old postmenopausal woman presented with abdominal distension and pain. Initial imaging, including ultrasound and CT, suggested an ovarian tumor. However, contrast-enhanced MRI identified a pyometra and pyocolpos complex, with malignant cytology detected in the drained fluid. Diagnostic exploratory laparotomy was necessitated due to extensive pyometra and pyocolpos precluding adequate diagnostic biopsy, with final histopathological confirmation of stage IIA1 squamous cell carcinoma of the cervix (FIGO 2018 criteria). Following the cervical cancer diagnosis, the patient underwent radical hysterectomy with bilateral salpingo-oophorectomy in late May 202, and received adjuvant radiotherapy post-discharge as further treatment.

**Clinical discussion:**

The coexistence of cervical cancer complicated by extensive pyometra and pyocolpos is a rare clinical condition that requires a comprehensive multidisciplinary diagnostic approach. For such complex presentations, a tripartite protocol—combining imaging continuity assessment, post-drainage cytomorphologic analysis, and histopathologic verification—is essential to circumvent diagnostic delays and sampling errors.

**Conclusion:**

①Postmenopausal pyometra cannot be excluded as a complication of gynecologic malignancies. High-grade squamous intraepithelial lesion (HSIL) and squamous cell carcinoma antigen (SCC) can directly extend proximally into the uterus. The most common clinical manifestations include pyometra and cervical stenosis. Emphasis should be placed on early detection and prevention of this condition. ②Combined human papillomavirus (HPV) testing and ThinPrep cytologic test (TCT) screening improve the detection rate of cervical lesions. However, in this patient, vaginal wall adhesions prevented cervical exposure, resulting in a false-negative finding and increasing the risk of missed diagnosis. ③Elevated serum levels of squamous cell carcinoma antigen (SCC-Ag) warrant heightened clinical vigilance. As a first-line serum biomarker for cervical cancer screening, SCC-Ag elevation may precede the onset of other clinical manifestations in affected patients. ④For cervical carcinomas with limited tumor size (maximal diameter <2 cm), a multimodal diagnostic integration incorporating serum tumor markers (SCC-Ag, CA125), advanced contrast-enhanced imaging (CT/MRI), and diagnostic surgical exploration may be considered. ⑤For early-stage cervical cancer patients complicated by uterovaginal abscess, a staged treatment approach should be adopted. And it’s also essential to balance the priorities of infection control and antitumor therapy.

## 1 Introduction

Cervical cancer is a significant public health issue worldwide, ranking as the fourth most common cancer among women. According to 2022 data, there are approximately 660,000 new cases and 350,000 deaths annually globally (1). The World Health Organization (WHO) aims to reduce the incidence of cervical cancer by 10% by 2030, by 70% by 2045, and ultimately by over 90% by 2120, necessitating global collaborative efforts that include strengthening HPV vaccination, screening, and treatment programs. Notably, despite a lower incidence compared to other cancers such as ovarian cancer, the mortality rate of cervical cancer is disproportionately high, highlighting the critical importance of early detection and preventive strategies.

Early diagnosis of cervical cancer remains clinically challenging in gynecologic oncology practice. Epidemiological studies reveal that diagnosed cases often present with locally advanced disease, mainly because of nonspecific early symptoms, insufficient screening compliance, and the limited sensitivity of existing detection methods. Although the widespread adoption of Human Papillomavirus (HPV) vaccination and cervical cancer screening programs has substantially reduced the incidence of the disease, early-stage cervical carcinoma frequently exhibits nonspecific clinical symptoms (2). Diagnostic accuracy is especially hindered in cases with concurrent anatomical abnormalities, such as vaginal adhesions, extensive pyometra and pyocolpos, significantly raising the risk of missed or incorrect diagnoses.

Current therapeutic strategies for cervical cancer chiefly encompass surgical resection, radiotherapy, and chemotherapy, with an integrated treatment plan that generally favors surgical management followed by adjuvant chemoradiotherapy (3). Radical hysterectomy (RH) combined with pelvic lymphadenectomy is the standard surgical procedure for early-stage cervical cancer, effectively removing lesions and prolonging patient survival (4). Although RH provides the benefit of extensive tumor removal, it’s highly invasive and may injure pelvic autonomic nerves, thereby elevating the risk of bladder and rectal dysfunction and adversely affecting postoperative quality of life.

Pyometra demonstrates a low incidence in the general population (0.1%-0.3%), but shows significantly higher prevalence among postmenopausal women (13.6%). Common etiologies include cervical stenosis, infections, malignant neoplasms, or radiation therapy (5). Notably, cervical cancer complicated by massive pyometra and concurrent pyocolpos remains exceptionally rare, with limited case reports documented in medical literature. These patients frequently receive misdiagnoses as ovarian tumors due to complex imaging manifestations, resulting in clinically significant treatment delays.

A study has found that postmenopausal spontaneous pyometra is significantly associated with gynecological malignancies, particularly endometrial cancer and cervical cancer (6). The pathogenesis involves malignant cervical lesions that precipitate anatomical obstruction at the cervical os, thereby impeding normal physiological drainage (7). Consequently, this obstruction facilitates the progressive accumulation of necrotic debris and inflammatory exudate within the uterine cavity, creating a conducive environment for polymicrobial infection and subsequent suppurative transformation, ultimately resulting in pyometra.

## 2 Case presentation

### 2.1 Clinical presentation

A 61-year-old postmenopausal woman (G2P2, natural menopause at age 56) presented with acute 4-day abdominal distension and pain. She remained afebrile without vaginal bleeding or discharge. Her medical history revealed no chronic comorbidities except for remote surgical history of nephrolithiasis. Unvaccinated for HPV with minimal alcohol use, she reported no family gynecologic malignancies. Initial transvaginal ultrasonography at a local hospital revealed a giant multiloculated pelvic cyst (252 × 110 × 105 mm) exhibiting a bilobed (“gourd-shaped”) configuration with heterogeneous echogenicity. Subsequent contrast-enhanced CT demonstrated a massive cystic uterine mass.

### 2.2 Diagnostic workup

① Physical examination: Vital signs are normal.

② Gynecological examination revealed a blind end of the vagina, but the cervix was not exposed. A huge mass can be palpated in the pelvic cavity, in the shape of a gourd, extending into the abdominal cavity. The boundary is clear, the range of motion is poor, and there is no tenderness.

③ Laboratory tests: Tumor markers: Serum tumor markers were significantly elevated: carbohydrate antigen 125 (CA125: 56.6U/mL), carbohydrate antigen 19-9 (CA19-9: 69.7U/mL), and squamous cell carcinoma antigen (SCC-Ag: 10.6ng/mL). The pronounced SCC-Ag elevation was consistent with cervical squamous cell carcinoma. Other tested parameters—including complete blood count, C-reactive protein (CRP), procalcitonin (PCT), human papillomavirus (HPV) DNA, ThinPrep cytologic test (TCT), and human epididymis secretory protein 4 (HE4)—yielded results within normal limits (**Supplementary Table 1**).

④ Imaging examination: Ultrasound examination of the uterus and adnexa reveals an abnormal mass extending from the abdominal cavity into the pelvic cavity, measuring approximately 211 × 93 mm (**Supplementary Figure 1**). Color Doppler imaging via transabdominal and transvaginal approaches indicates a large mass occupying the pelvic and abdominal regions. However, the exact nature of the mass could not be definitively characterized by color Doppler ultrasound. Further pelvic magnetic resonance imaging (MRI) revealed a large cystic lesion in the pelvic cavity, approximately 106 × 97 × 225 mm in size, with possible considerable fluid accumulation within the uterine cavity and vagina (**Supplementary Figures 2**-**6**).

### 2.3 Therapeutic intervention

**Figure 1:**
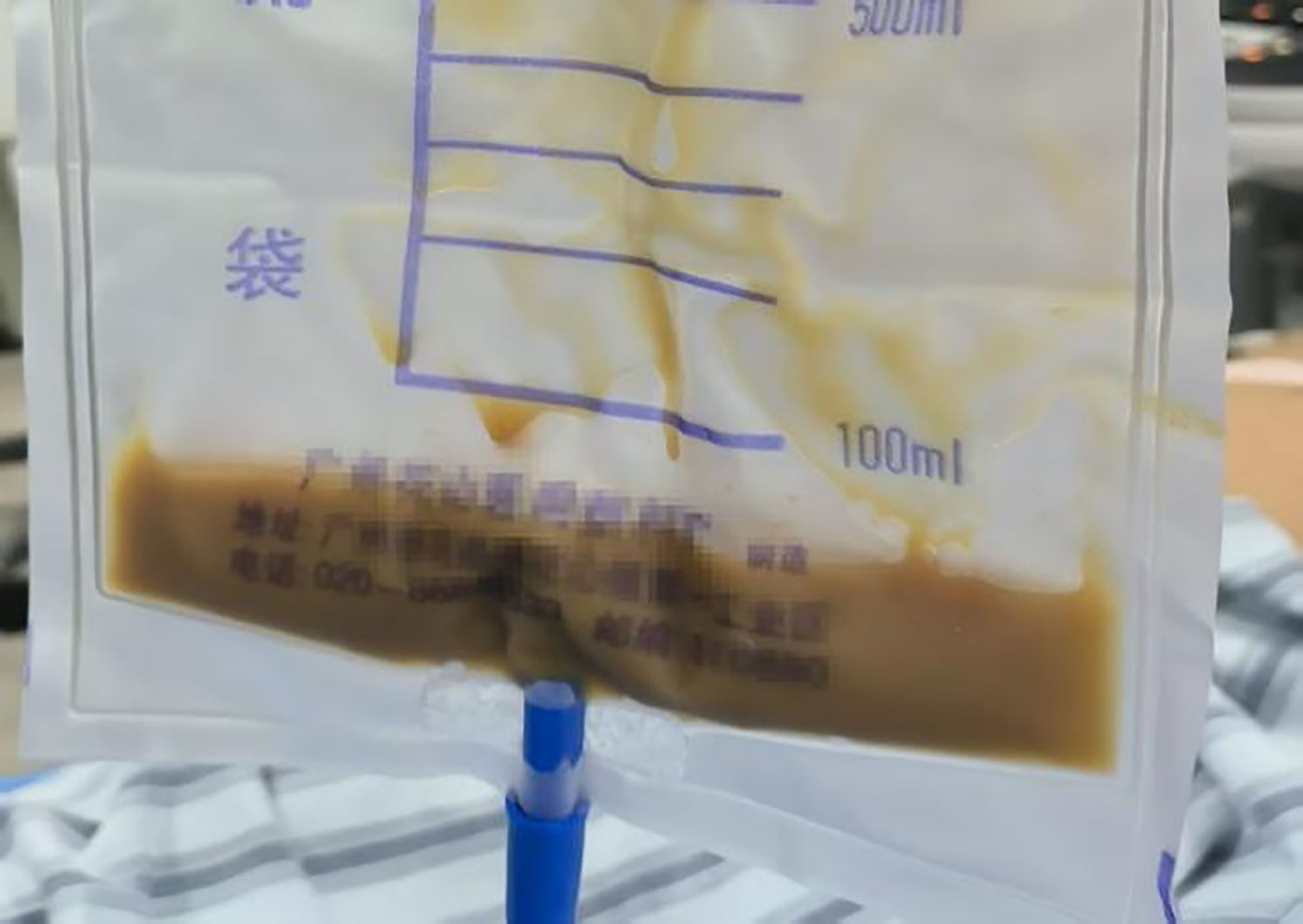
Purulent fluid drained from the uterine cavity.

Pelvic MRI revealed substantial pyometra and pyocolpos. Therefore, we performed ultrasound-guided transvaginal drainage, successfully aspirating approximately 900 mL of light brown purulent fluid (**Figure 1**). Intraoperatively, the patient acutely developed lower abdominal pain with associated rectal tenesmus, pallor, and diaphoresis. Hemodynamic parameters remained stable (HR 74 bpm, RR 20/min, BP 108/68 mmHg). The constellation of clinical findings supported a diagnosis of post-decompression vasovagal reflex. The vasovagal symptoms resolved completely following intravenous crystalloid administration and appropriate analgesic intervention. Postoperative management comprised daily uterine cavity irrigation with 0.5% metronidazole solution, drainage tube removal on postoperative day 3, and intravenous antimicrobial therapy (ceftriaxone 2g qd plus ornidazole 500mg bid for 7 days). Postoperative evaluation included comprehensive diagnostic assessments to monitor treatment response and disease progression.

**Figure 2:**
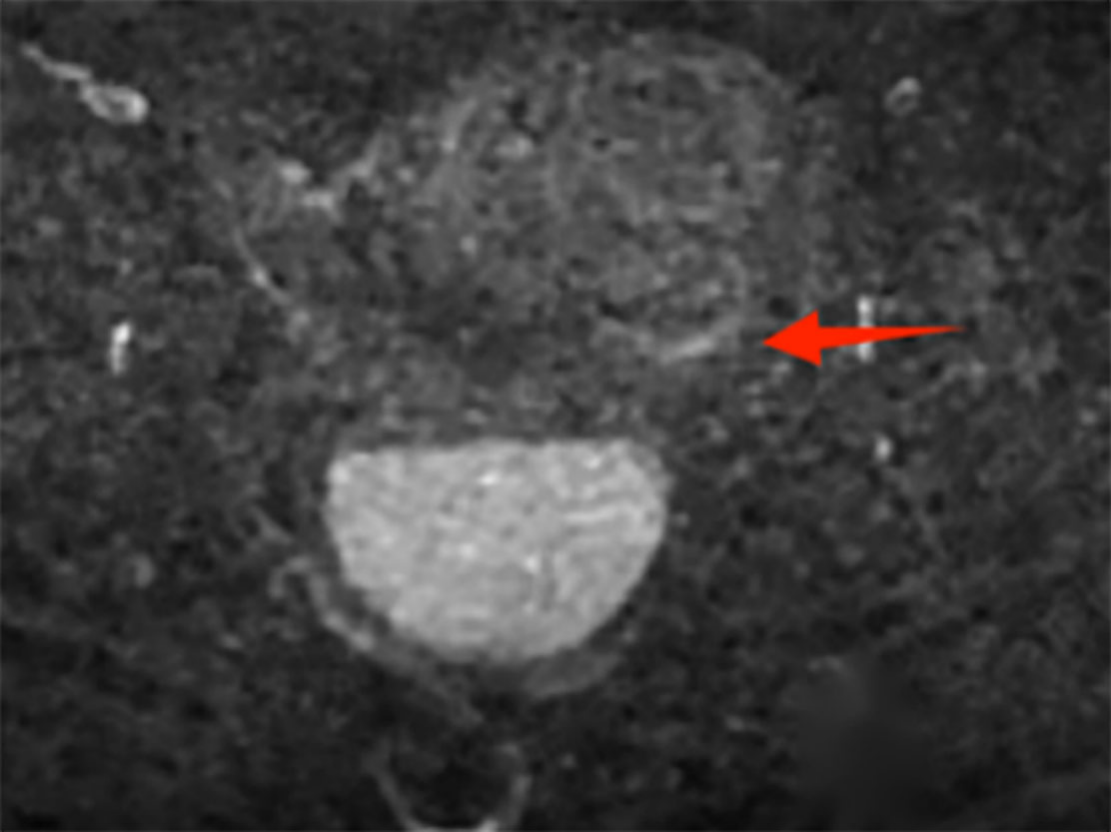
Post-drainage follow-up MRI T2-weighted imaging demonstrates stratification of cystic fluid; the left lateral cervical wall shows focal mild diffusion restriction, with hyperintense signal on DWI.

The postoperative laboratory and imaging examination results are as follows: ① Microbiological examination: Both aerobic and anaerobic bacteria cultures in the drainage fluid were negative. ② Color Doppler ultrasound of the uterine adnexa indicates: enlarged uterine volume accompanied by signs of uterine cavity effusion (**Supplementary Figure 8**). ③ Contrast-enhanced pelvic MRI demonstrated localized mild diffusion restriction involving the left lateral cervical wall, suggestive of early stromal infiltration. ④ Cytology revealed malignant squamous cells. (**Supplementary Figure 9**; **Figure 2**).

However, definitive histopathological confirmation through cervical biopsy proved impossible due to severe pyometra and vaginal wall adhesions. After completing rigorous multidisciplinary evaluation and obtaining fully documented informed consent through comprehensive family discussions, the decision was made to proceed with diagnostic laparotomy.

The patient underwent radical hysterectomy with bilateral salpingo-oophorectomy and 2-cm vaginal resection. Intraoperative inspection revealed intact endometrial and vaginal mucosa, with successful lysis of vaginal adhesions and reconstruction of vaginal patency. Final pathology revealed cervical squamous cell carcinoma (moderately differentiated) with 0.7 mm stromal invasion, 3 mm horizontal spread, and <1 mm vaginal margin involvement, accompanied by High-Grade Squamous Intraepithelial Lesion (HSIL/CIN 3) (**Supplementary Figure 11**). Immunohistochemistry supported HPV-associated carcinogenesis (diffuse block-type p16+), high proliferation (Ki-67>90%), and squamous differentiation (CK5/6+/pan-CK+) with intact basement membrane (type IV collagen+) (**Supplementary Figure 12**). According to FIGO 2018 criteria, the diagnosis was stage IIA1 cervical cancer. The patient recovered well and was discharged to undergo adjuvant radiotherapy.

The patient is one year post-surgery, with a postoperative review conducted in February 2025. Laboratory and imaging examinations showed no abnormalities, indicating good recovery. During approximately six months of adjuvant radiochemotherapy, the patient experienced increased vaginal discharge and performed self-administered vaginal irrigation without significant discomfort. Recently, after sexual activity, the patient reported vaginal pain and discomfort, accompanied by a small amount of bleeding, suggesting possible local vaginal mucosal lesions or adhesions.

Complete blood count revealed leukocytosis, indicative of an inflammatory response or infection. Tumor marker SCC-Ag was 0.7 ng/mL, within the normal range, suggesting no increased risk of tumor recurrence. Gynecological examination showed smooth vaginal mucosa with mild adhesions at the apex. After adhesiolysis, a few pinpoint bleeding spots were observed, which may represent minor vascular rupture or microbleeding related to local adhesions. Abdominal contrast-enhanced CT demonstrated hepatic hemangiomas and cysts, decreased right renal volume, and renal cysts on the left side. These findings are consistent with pre-existing benign lesions or cysts, with no evidence of malignant transformation.

Overall, the patient is recovering well postoperatively, with no signs of tumor recurrence (normal SCC-Ag, no mass evident on imaging). Nonetheless, vigilance is advised regarding local vaginal inflammation and adhesion issues. Anti-inflammatory treatment and standardized follow-up are recommended, along with avoiding activities such as vaginal irrigation that may disrupt the local microenvironment. The benign lesions in the liver and kidneys require multidisciplinary monitoring to exclude any progression risk.

## 3 Discussion

The diagnosis of cervical cancer accompanied by pyometra and pyocolpos presents significant challenges. Studies have shown that only approximately 10% of cervical cancer patients manifest the classic clinical trial of vaginal purulent discharge, postmenopausal bleeding, and abdominal pain (8). The cervical mass in cervical cancer patients blocks the cervical canal, causing pyometra that cannot drain naturally through the vagina, thus covering up the typical purulent discharge. The accumulation of purulent material due to infectious lesions closely overlaps with the mass effect of malignant cervical tumors in clinical and radiological features. This morphological similarity can lead to diagnostic challenges, potentially resulting in misdiagnosis or delayed treatment.

There is a report of cases involving spontaneous rupture of pyometra caused by cervical cancer (9). Among 26 patients, all but one were postmenopausal elderly women, and all exhibited clinical features of generalized peritonitis. In these cases, preoperative diagnosis of perforated pyometra was rarely made. Only one case described the CT features of the perforated pyometra. Therefore, in postmenopausal women presenting with abdominal pain combined with vaginal purulent discharge or bleeding, a comprehensive evaluation is recommended. This includes a gynecological examination focusing on cervical morphology, serum tumor marker testing (such as SCC-Ag and CA125), imaging studies (ultrasound or MRI to assess cervical and uterine cavity continuity), and tissue biopsy. When imaging indicates intrauterine fluid accumulation, cytological analysis of the drained fluid can serve as a valuable auxiliary diagnostic method. It is also important to note that accumulated purulent fluid may contaminate pathological specimens and interfere with the identification of tumor cells.

Management of early-stage cervical cancer complicated by uterovaginal abscessors necessitates a staged therapeutic approach. Initially, infected fluid accumulation should be addressed through drainage to control the infection. Once infection stabilization is achieved, definitive radical surgical resection is indicated. Drainage procedures must adhere to standardized protocols: employing stepwise decompression with controlled drainage rates (≤50 mL/min) to minimize the risk of post-decompression vasovagal reflex. In cases with a higher contamination risk, negative pressure wound therapy is recommended. Perioperative antibiotic management is critical; regardless of initial infection markers (e.g.leukocyte count, C-reactive protein) or the results of oxygen/anaerobic cultures from the drainage fluid, all patients should receive prophylactic antibiotics as per standard guidelines. This strategy aims to mitigate potential subclinical infections and effectively prevent postoperative pelvic effusion, uterine rupture, peritonitis, septic shock, and sepsis (10–12).

Additionally, management requires balancing infection control and anti-tumor therapy priorities: initiating radiochemotherapy too early may trigger or exacerbate pyometra risk, while delaying treatment can lead to tumor progression. A case report describes a 102-year-old woman who presented with a one-week history of foul-smelling, coffee-ground-like vaginal discharge, anorexia, and generalized weakness. Pelvic examination revealed signs of endometrial and vaginal inflammation. Multidetector computed tomography (MDCT) showed a large cystic renal pelvis lesion and mild dilation of the intrahepatic bile ducts on the left side. Cervical biopsy confirmed a high-grade intraepithelial lesion (CIN3), leading to a diagnosis of stage IB cervical carcinoma. The patient received localized radiotherapy, and ten days post-treatment, she was readmitted due to decreased appetite, somnolence, vaginal bleeding with purulent discharge, and leukocytosis. Serum CA-125 level was elevated to 1598 U/mL. The diagnosis was pyometra secondary to tumor regression after radiotherapy. Based on this case, we recommend vigilant monitoring for the accumulation of purulent fluid during cervical cancer treatment, and implementing proactive measures to prevent the development of pyometra, thereby reducing the risk of infectious complications.

This study reports a critical gynecologic oncological emergency: cervical cancer accompanied by pyometra and pyocolpos. The diagnosis presents a significant challenge because of the substantial overlap between the effects of the tumor mass and the symptoms of infection, compounded by the impossibility of obtaining a biopsy for pathological examination due to vaginal adhesions. The treatment dilemma involves balancing prompt infection control with timely oncologic therapy, with no current systematic solution available. International evidence-based guidelines, such as NCCN and ESGO, do not provide specific recommendations for this clinical scenario. Based on real-world clinical data, this study is the first to develop and validate a standardized management framework that integrates diagnostic pathways and staging treatment strategies. The proposed approach aims to offer practical guidance for clinicians, fill the evidence gap in this field, reduce misdiagnosis, prevent treatment-related complications, and improve short-term patient outcomes.

This study is a case series that provides important insights into gynecological emergency tumors, specifically cervical cancer complicated by uterine pyometra and vaginal pyometra, while also emphasizing the necessity of establishing standardized protocols. However, several limitations should be acknowledged: ①As a single-center case series, the management approach may not be applicable to all patient subgroups. ②Data collection being retrospective carries risks of selection and information bias, and it did not include dynamic assessments of pus microbiological load or tumor markers. ③The 2017 Querleu-Morrow classification introduced new categories for radicular hysterectomy (13)—including Type A (Limited Radical Hysterectomy), Type B (Resection of the Paracervix at the Ureter), Type C1 (Nerve-preserving Radical Hysterectomy), Type C2 (Radical Hysterectomy without Autonomic Nerve Preservation), and Laterally Extended Resection. Considering the recovery of bladder and rectal functions, future surgical procedures should favor nerve-sparing radical hysterectomy techniques to preserve autonomic nerve function and optimize outcomes. ④Ovarian and vaginal shortening due to cervical occlusion restrict the extent of surgical resection— in this case, vaginal excision was only 2cm, whereas the standard is 3-4cm. ⑤A one-year follow-up is inadequate to thoroughly evaluate long-term recurrence risks.

Despite these limitations, this study provides preliminary clinical insights into cervical cancer complicated by uterine and vaginal pyometra and highlights the urgent need to conduct prospective cohort studies to develop standardized treatment protocols.

## 4 Conclusions

This study reports a special case of a 61-year-old postmenopausal woman with stage IIA1 cervical squamous cell carcinoma initially misdiagnosed as an ovarian tumor due to abdominal distension. This case provides three key contributions to gynecologic oncology practice: First, it elucidates the atypical clinical presentation of cervical cancer in the setting of anatomical distortion (vaginal atresia). Second, it validates the diagnostic utility of a multimodal approach integrating tumor markers (SCC-Ag, CA125),advanced imaging (MRI),and surgical exploration when conventional methods fail. Third, it offers novel therapeutic insights for malignancy-associated pyometra management, particularly regarding drainage techniques and timing of definitive surgery.

These findings underscore the importance of maintaining high suspicion for malignancy in postmenopausal women with pelvic masses, even without typical symptoms, and the necessity of employing comprehensive diagnostic strategies when anatomical limitations exist. Future prospective studies should focus on optimizing standardized protocols for such complex scenarios to improve diagnostic accuracy and therapeutic outcomes.

## Data Availability

The original contributions presented in the study are included in the article/**Supplementary Material**. Further inquiries can be directed to the corresponding author.

## Supplementary material

Supplementary Table 1 Serum biomarkers at admission.

Supplementary Figure 1 Initial pelvic ultrasound on admission demonstrating a giant cystic mass in the abdominopelvic cavity.

Supplementary Figure 2 Pelvic MRI on admission revealed a bilobed (“gourd-shaped”) mass with markedly enhanced uniform wall thickening (maximum thickness: 6 mm in upper segment) and non-enhanced cystic content.

Supplementary Figure 3 Admission MRI (T1-weighted imaging) demonstrating slightly hyperintense signal intensity within the cystic fluid.

Supplementary Figure 4 T2-weighted MRI at initial evaluation demonstrates markedly hyperintense signal intensity.

Supplementary Figure 5 Initial MRI shows hyperintense ADC signal, high-tension cystic fluid with sediment, and marked bladder compression.

Supplementary Figure 6 Initial diffusion-weighted MRI demonstrates markedly hyperintense signal.

Supplementary Figure 7 Purulent fluid drained from the uterine cavity.

Supplementary Figure 8 Post-drainage ultrasound demonstrates uterine enlargement with minimal residual uterine and vaginal fluid collection.

Supplementary Figure 9 Post-drainage MRI: ADC map demonstrates fluid layering with focal hypointense signal in the left cervical wall.

Supplementary Figure 10 Post-drainage follow-up MRI T2-weighted imaging demonstrates stratification of cystic fluid; the left lateral cervical wall shows focal mild diffusion restriction, with hyperintense signal on DWI.

Supplementary Figure 11 Histopathological results demonstrate squamous cell carcinoma.

Supplementary Figure 12 Immunohistochemical staining image of the lesion site.
