# Supplementary figures and images for: Case Report: Cervical cancer masquerading as ovarian tumor: diagnostic challenges in a case with severe pyometra-pyocolpos complex
Source: Front Oncol. 2025 Aug 27;15:1647366. doi: 10.3389/fonc.2025.1647366 (PMC12421625; doi:10.3389/fonc.2025.1647366)

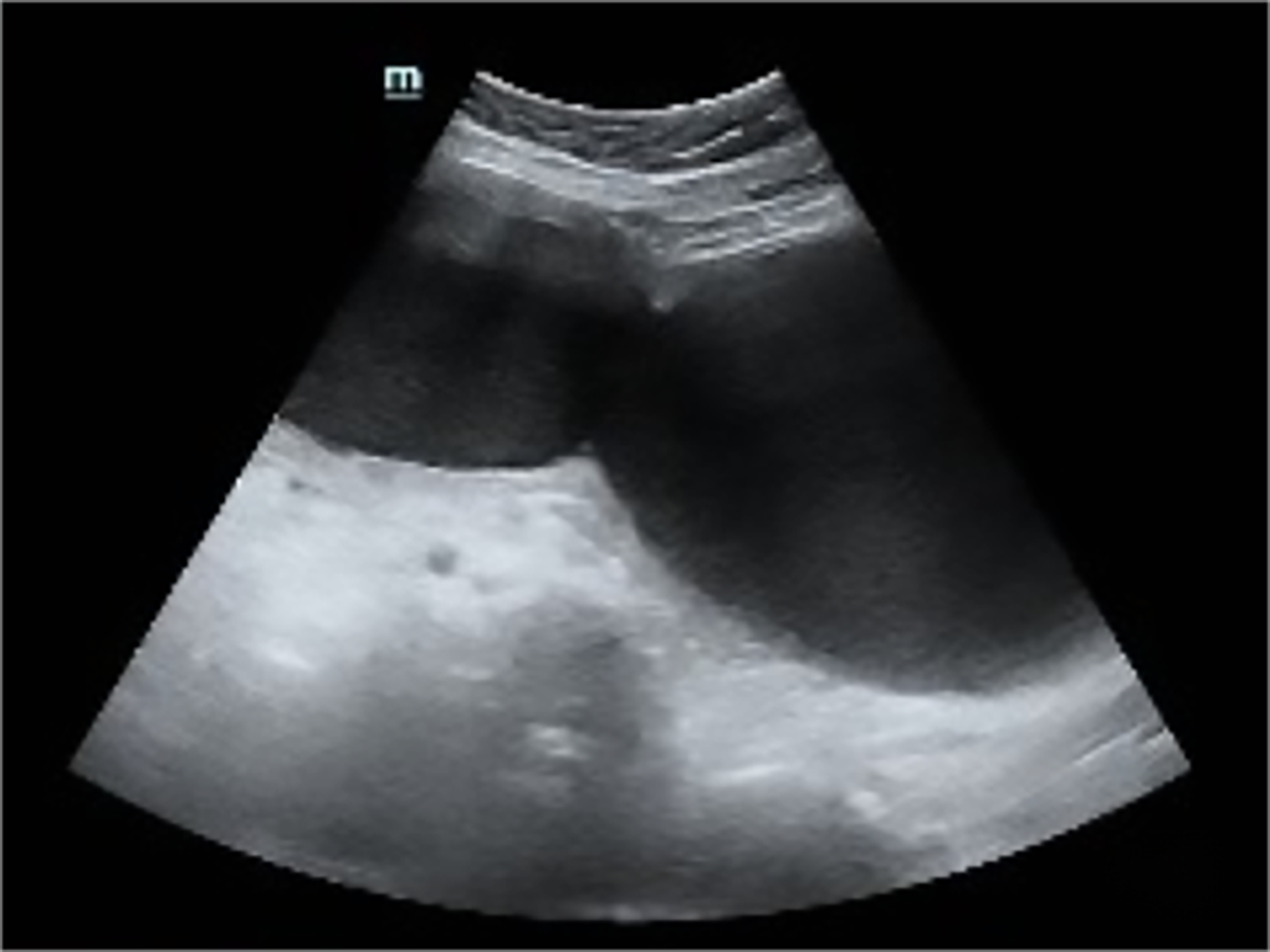

Supplement: Supplementary Table 1 — Serum biomarkers at admission. [file Image1.tif]

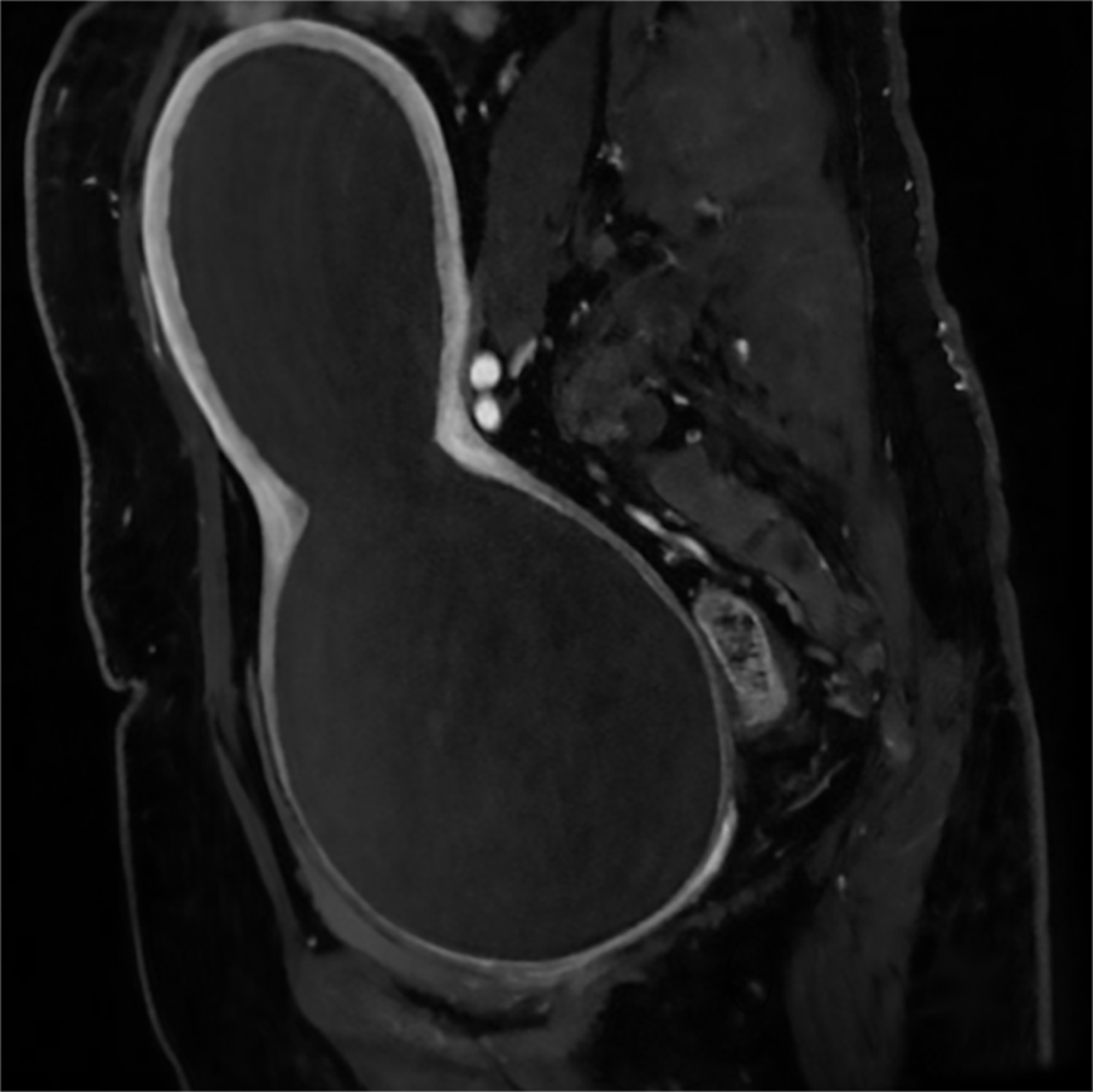

Supplement: Supplementary Figure 1 — Initial pelvic ultrasound on admission demonstrating a giant cystic mass in the abdominopelvic cavity. [file Image2.tif]

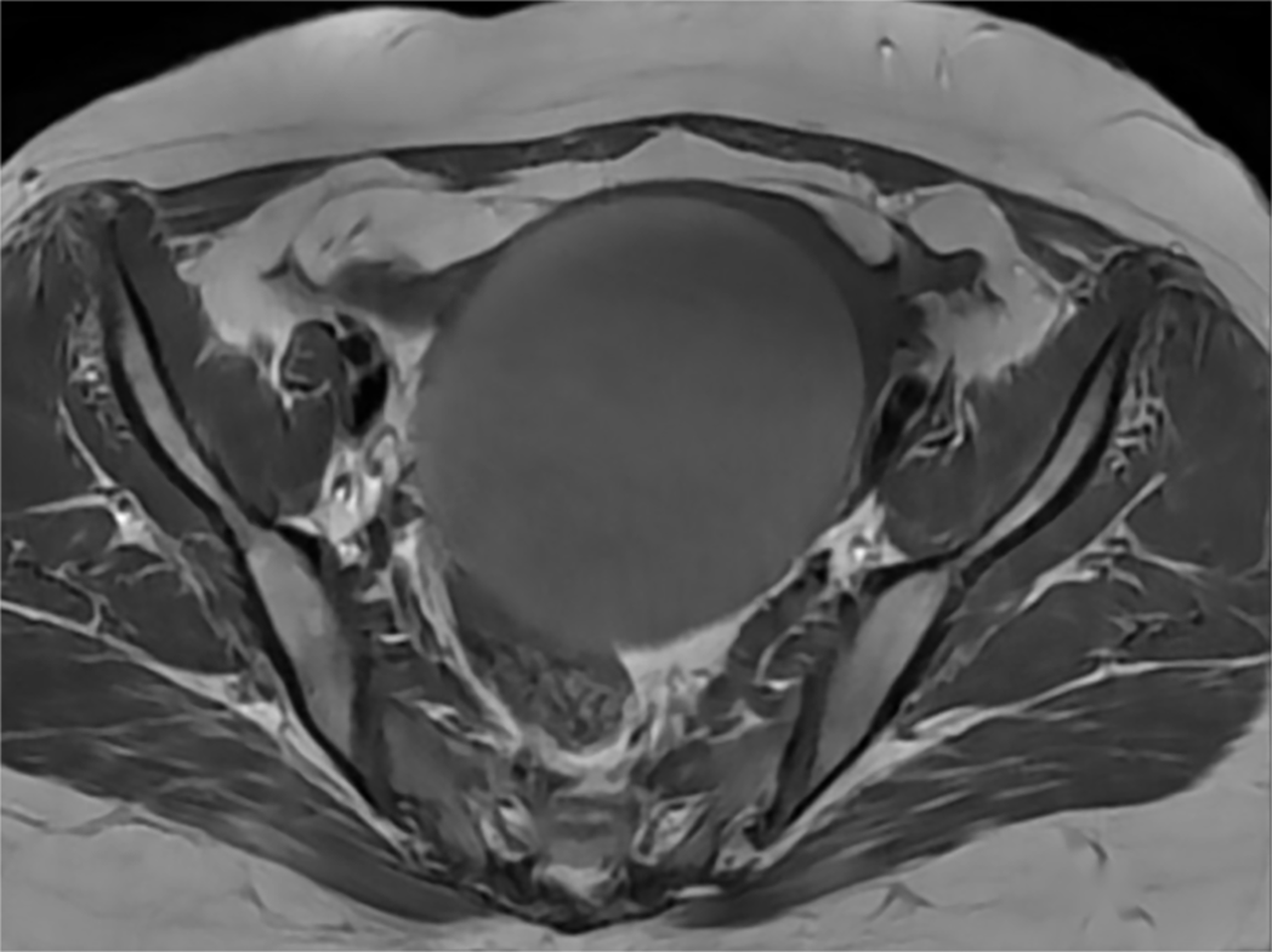

Supplement: Supplementary Figure 2 — Pelvic MRI on admission revealed a bilobed (“gourd-shaped”) mass with markedly enhanced uniform wall thickening (maximum thickness: 6 mm in upper segment) and non-enhanced cystic content. [file Image3.tif]

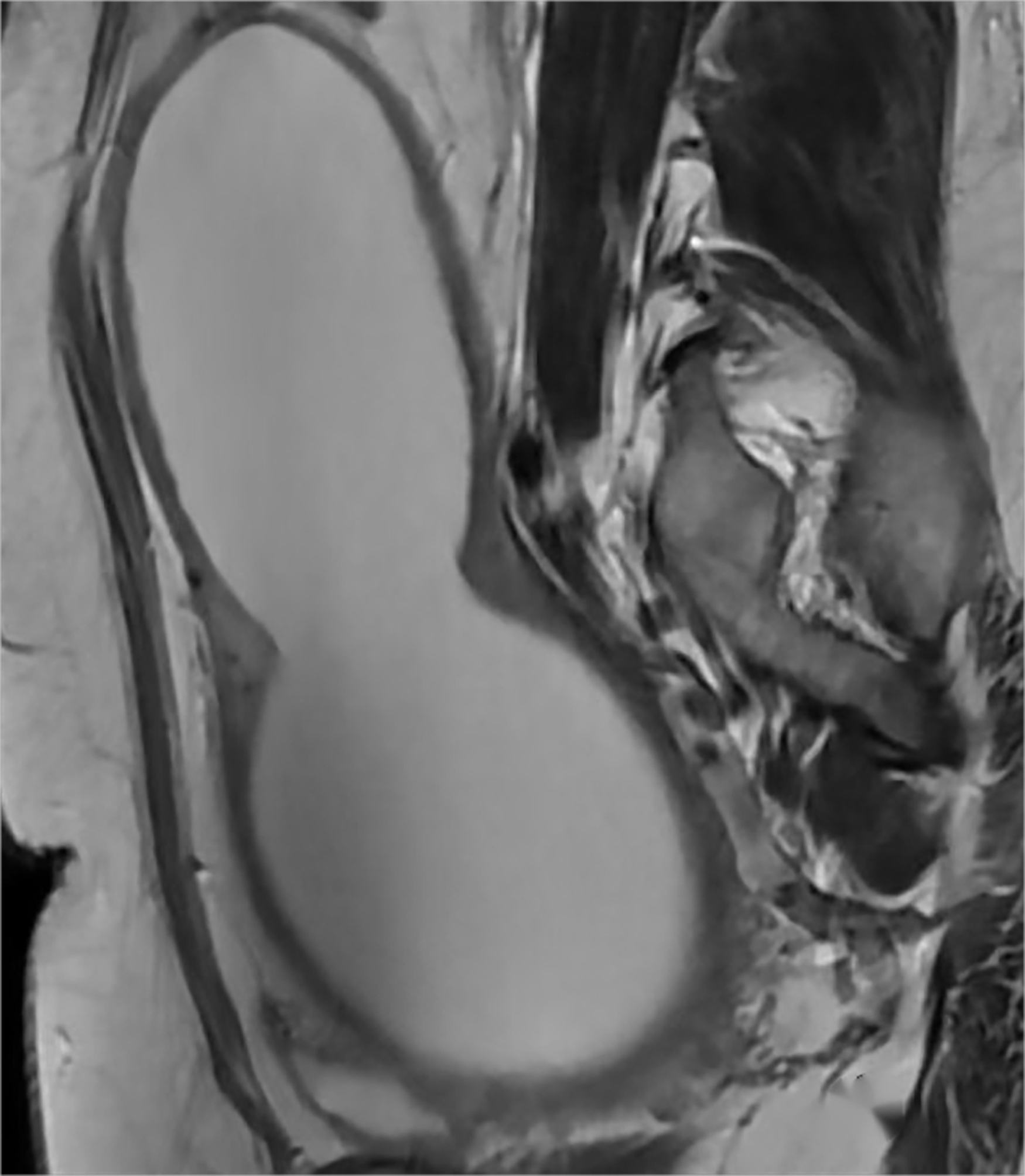

Supplement: Supplementary Figure 3 — Admission MRI (T1-weighted imaging) demonstrating slightly hyperintense signal intensity within the cystic fluid. [file Image4.tif]

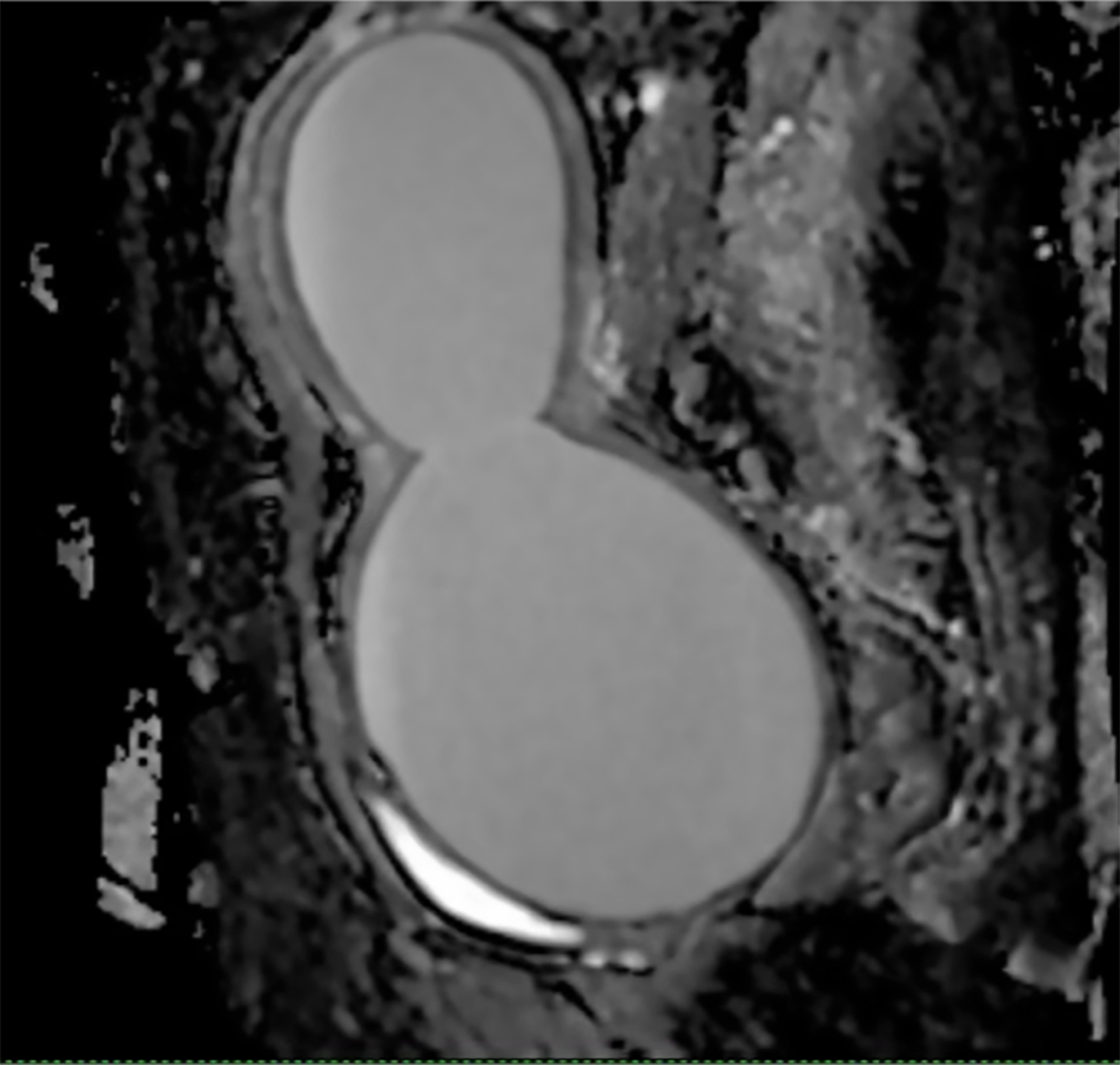

Supplement: Supplementary Figure 4 — T2-weighted MRI at initial evaluation demonstrates markedly hyperintense signal intensity. [file Image5.tif]

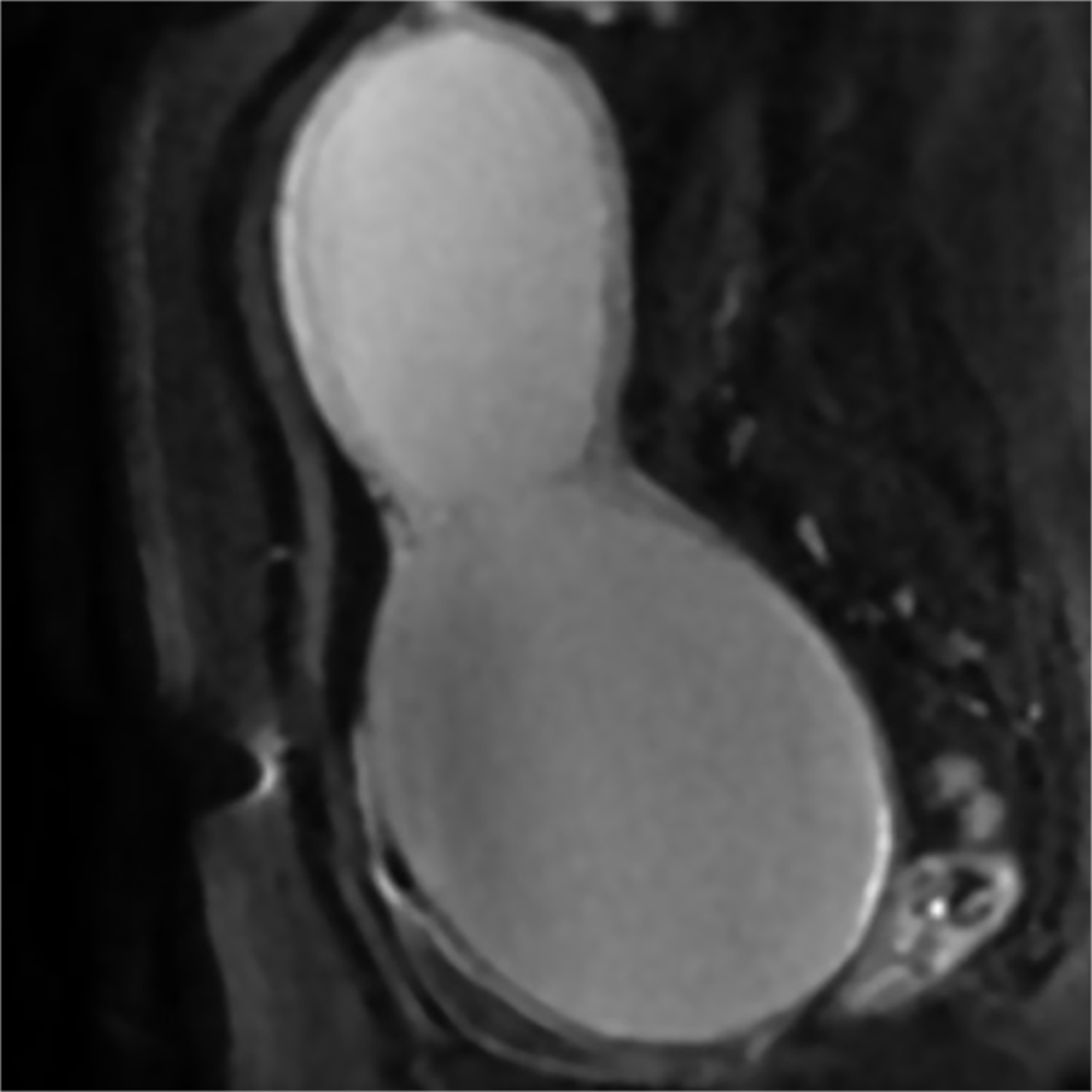

Supplement: Supplementary Figure 5 — Initial MRI shows hyperintense ADC signal, high-tension cystic fluid with sediment, and marked bladder compression. [file Image6.tif]

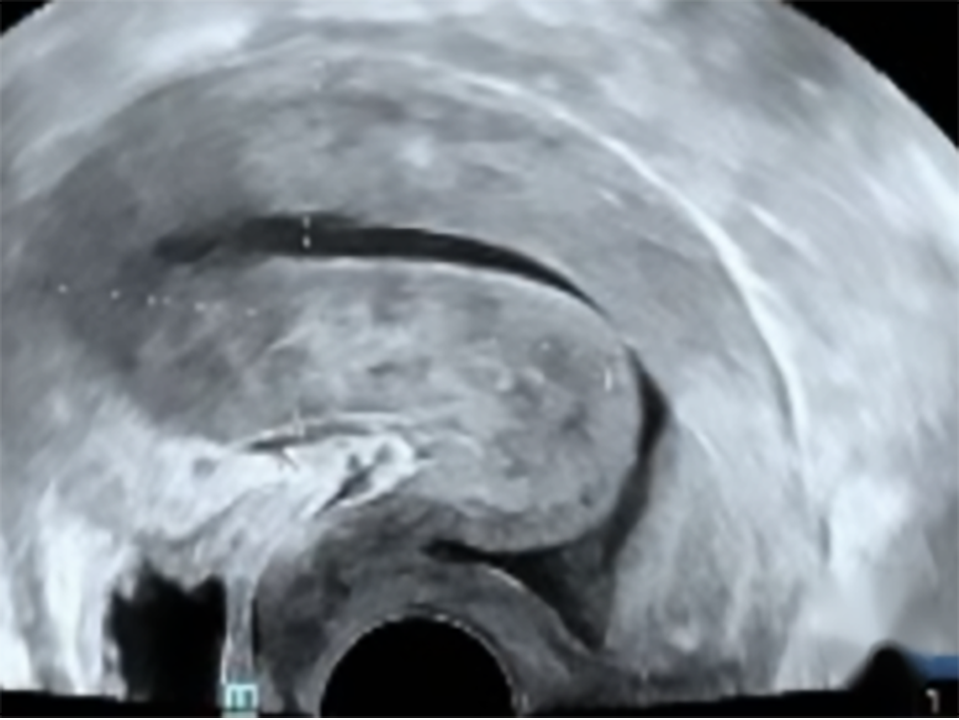

Supplement: Supplementary Figure 6 — Initial diffusion-weighted MRI demonstrates markedly hyperintense signal. [file Image7.tif]

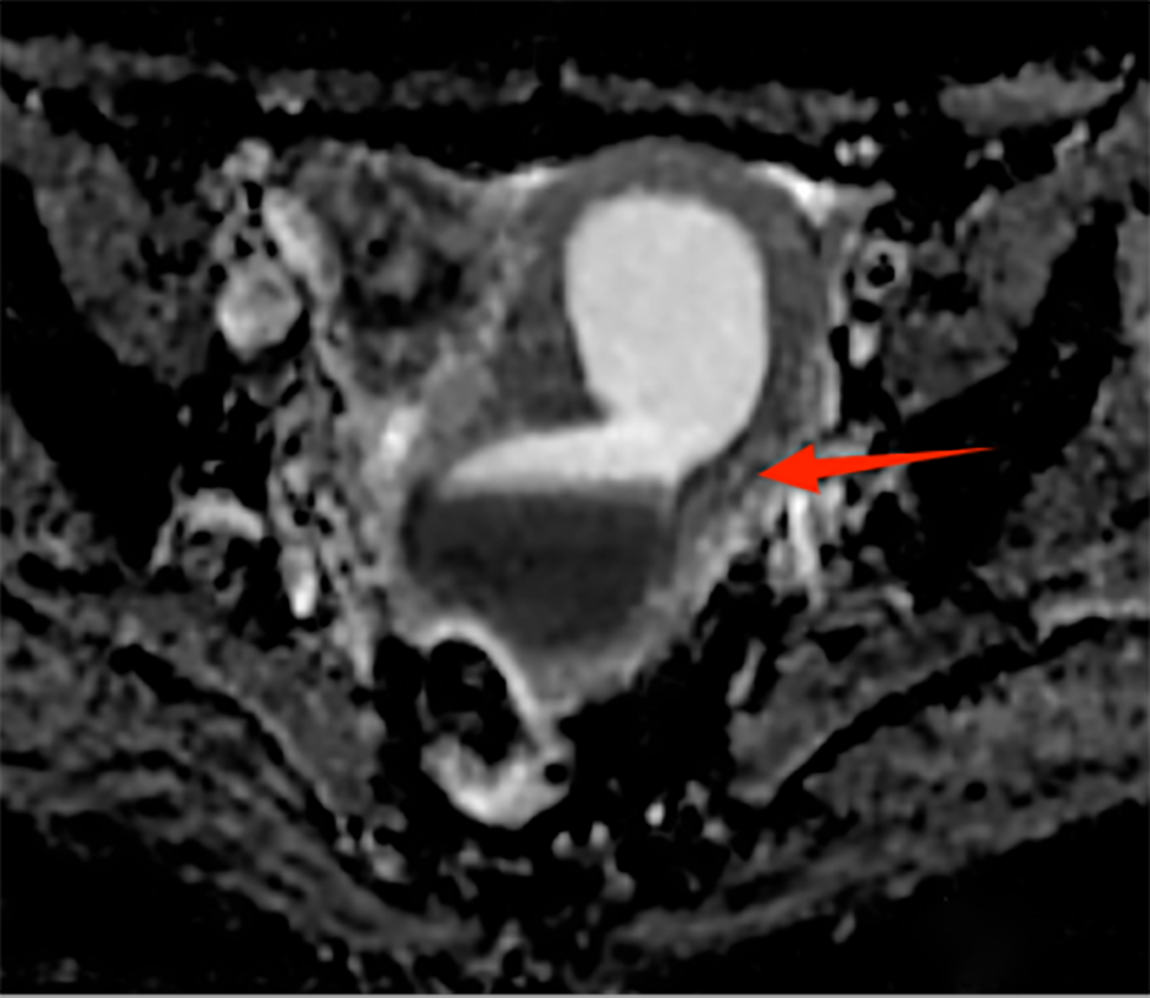

Supplement: Supplementary Figure 7 — Purulent fluid drained from the uterine cavity. [file Image8.tif]

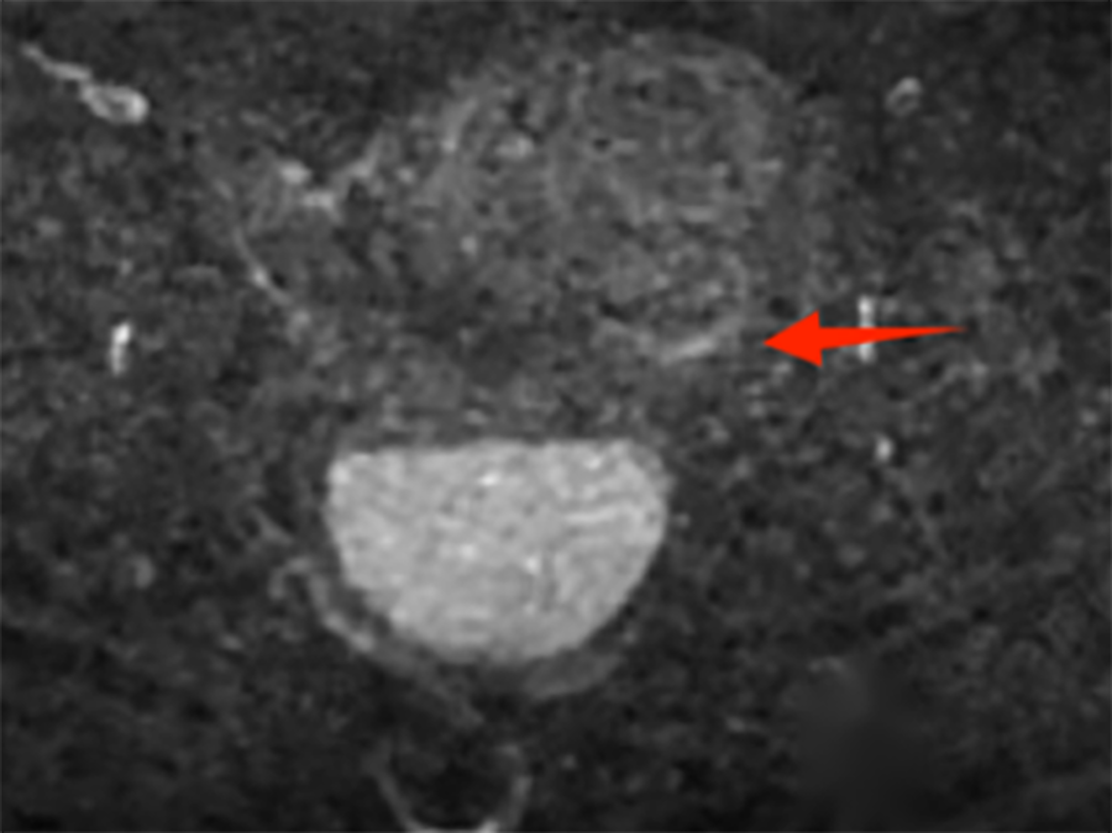

Supplement: Supplementary Figure 8 — Post-drainage ultrasound demonstrates uterine enlargement with minimal residual uterine and vaginal fluid collection. [file Image9.tif]

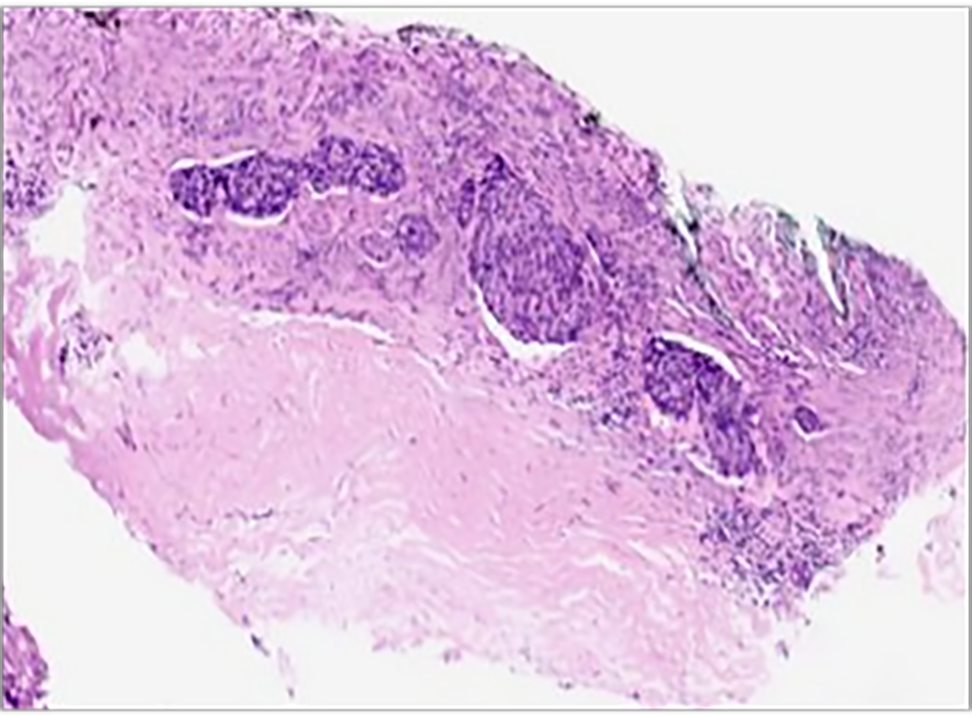

Supplement: Supplementary Figure 9 — Post-drainage MRI: ADC map demonstrates fluid layering with focal hypointense signal in the left cervical wall. [file Image10.tif]

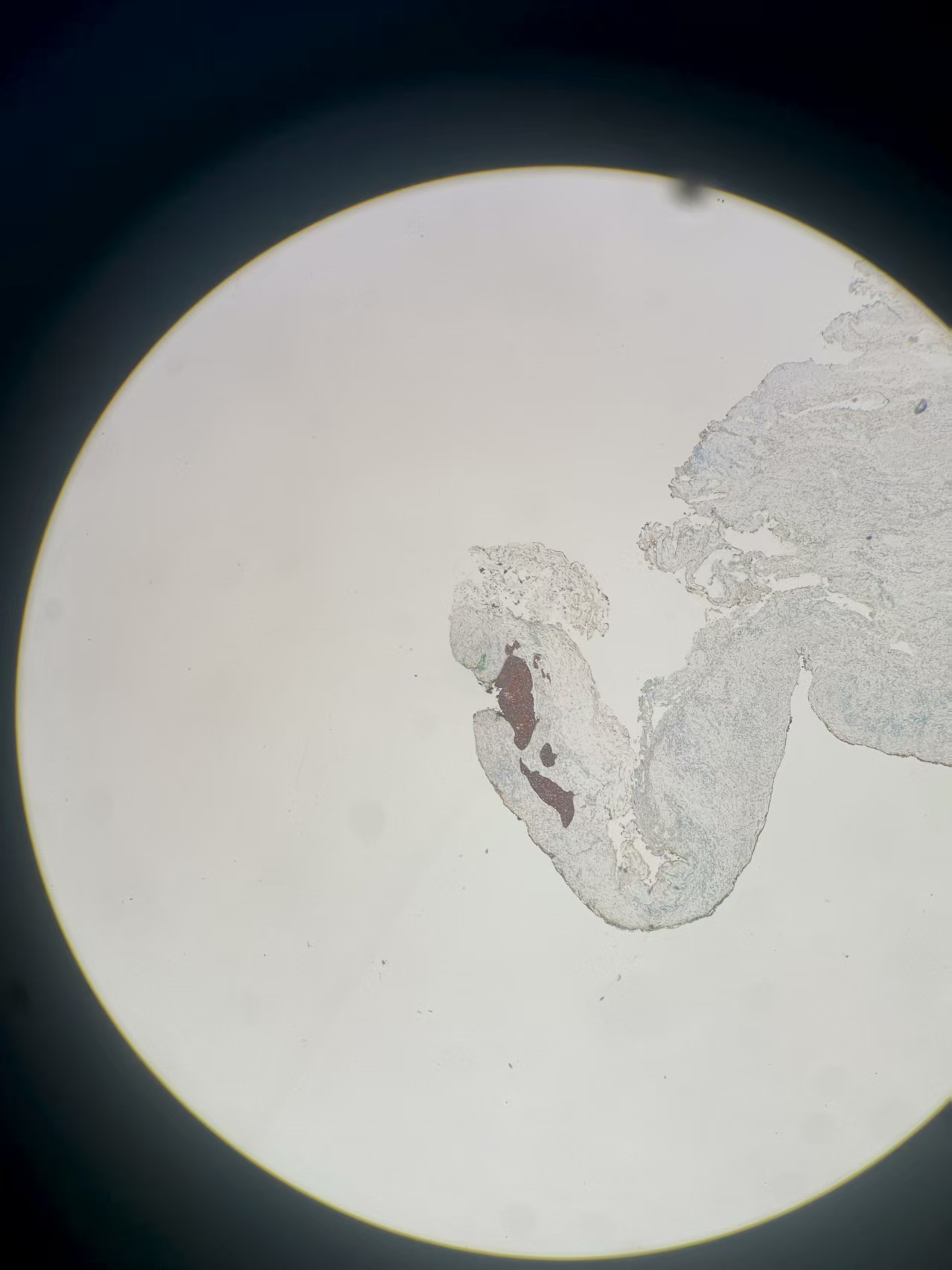

Supplement: Supplementary Figure 10 — Post-drainage follow-up MRI T2-weighted imaging demonstrates stratification of cystic fluid; the left lateral cervical wall shows focal mild diffusion restriction, with hyperintense signal on DWI. [file Image11.tif]

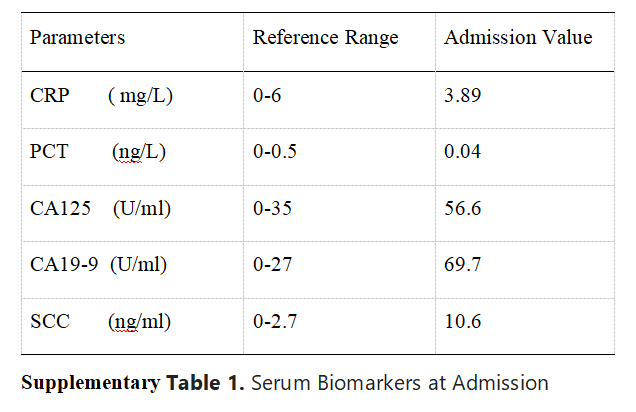

Supplement: Supplementary Figure 11 — Histopathological results demonstrate squamous cell carcinoma. [file Image12.png]
